# Accuracy of self-collection versus cervical sampling for the molecular diagnosis of *Chlamydia trachomatis* and *Neisseria gonorrhoeae* in women attending gynecological services

**DOI:** 10.61622/rbgo/2025rbgo31

**Published:** 2025-07-15

**Authors:** Gabriela Vasco, Cecilia Cruz, Paterson Peñaherrera, Katherine Tipán, Sandy Pila, Karol Guzmán, Marisol Cabascango, Katherine Logacho, Patricio Jácome

**Affiliations:** 1 Universidad Central del Ecuador Facultad de Ciencias Médicas Quito Ecuador Facultad de Ciencias Médicas, Universidad Central del Ecuador, Quito, Ecuador.; 2 Unidad de Genética y Molecular Instituto Ecuatoriano de Seguridad Social Hospital de Especialidades Carlos Andrade Marín Quito Ecuador Unidad de Genética y Molecular, Hospital de Especialidades Carlos Andrade Marín, Instituto Ecuatoriano de Seguridad Social, Quito, Ecuador.; 3 Servicio de Atención Integral para Adolescentes Hospital Gineco-Obstétrico Isidro Ayora Ministerio de Salud Quito Ecuador Servicio de Atención Integral para Adolescentes, Hospital Gineco-Obstétrico Isidro Ayora, Ministerio de Salud, Quito, Ecuador.

**Keywords:** Chlamydia trachomatis, Neisseria gonorrhoeae, Gonorrhea, Sexually transmitted diseases, Women, Prevalence

## Abstract

**Objective:**

Successful measures to address the increasing prevalence of sexually transmitted infections (STIs) require practical and accessible education and detection programs.

**Methods:**

The ability to detect *Chlamydia trachomatis* and *Neisseria gonorrhoeae* may be hindered by a lack of adherence to proper cervical sampling. To address this, we compared the sensitivity and specificity of self-obtained samplings, such as self-collection and first-catch urine samples, to cervical samples taken by a practitioner using the nucleic acid amplification test (NAAT) cobas^®^ 4800 for *C. trachomatis* and *N. gonorrhoeae* in 244 women attending gynecological services in Quito, Ecuador. Regardless of sampling method, only 12 patients tested positive for *C. trachomatis* (4.9% prevalence, 95% CI 2.8 to 8.4%), and no samples tested positive for *N. gonorrhoeae*.

**Results:**

The analysis revealed that self-collection was 100% sensitive (95% CI 66.4% to 100.0%) and 100% specific (95% CI 98.25%-100%), and first-catch urine was 90% sensitive (95% CI 55.5% to 99.8%), and 99% specific (95% CI 96.5% to 99.9%) compared to cervical brushing for the detection of *C. trachomatis*. No symptoms were associated with a positive *C. trachomatis* result, highlighting the need for testing even in asymptomatic patients. Furthermore, having a stable intimate relationship in the past year was associated with a negative result for *C. trachomatis* (χ^2^ 14.01, *p* < 0.001).

**Conclusion:**

This study demonstrates the feasibility and reliability of self-collection and first-catch urine samples as alternative methods for detecting *Chlamydia trachomatis* and has practical implications for improving STI detection and management programs.

## Introduction

One million new curable sexually transmitted infection (STI) cases, including chlamydia, gonorrhea, syphilis, and trichomoniasis, occur daily.^(1,2)^ The promotion of causative management has significantly improved the accuracy of STI diagnosis through diagnosis of the etiologic agents and implementation of simplified protocols in clinics.^(2,3)^ In contrast, syndromic management only has an accuracy rate of less than 30% for vaginal discharge syndrome.^(4)^ Often, syndromic management excludes up to 80% of patients, mainly asymptomatic.^(5,6)^

For women, a gynecological examination by a professional can help detect visible lesions related to vulvovaginal diseases and significantly improve the diagnosis process. Particularly for *Chlamydia trachomatis* and *Neisseria gonorrhoeae*, cervical brushing is the recommended sample collection technique. However, gynecological practitioners may discourage a full speculum examination due to lack of clinical symptoms, such as abdominal pain or vaginal discharge, or if there is no indication of a Papanicolaou test, which reduces the chances of detecting asymptomatic STIs.^(7)^ Blake et al.^(8)^ demonstrated that using self-obtained vaginal swabs in women had more acceptability and was significantly more cost-effective in detecting new cases of *C. trachomatis*. It is essential to note that the presence or absence of symptoms attributable to STIs doesn't prevent the development of disease complications for patients, such as infertility, ectopic pregnancy, and inflammatory pelvic disease in women, while newborns can suffer from ophthalmia neonatorum, pneumonia, or pre-term birth.^(9)^

Various regional and sexual risk factors impact the prevalence of *C. trachomatis* and *N. gonorrhoeae*, especially when including asymptomatic cases.^(6,10)^ Du et al.^(11)^ determined the long-term age-standardized incidence rates (ASR) for STIs globally from 1990 to 2019. Notably, there was an increasing global tendency in ASR from 2010-2019. American regions had the highest growing prevalence of syphilis, chlamydia, and gonorrhea.^(11)^ Similarly, Rowley et al.^(1)^ also demonstrated that the American region had the highest estimated prevalences of 7.6% in 2012 and 7% in 2016 for chlamydia, and the third highest for gonorrhea with 0.8 and 0.9%, respectively, when compared to other World Health Organization regions.^(1)^ Moreover, Latin America has the highest prevalence of *C. trachomatis* worldwide and a lower than 1% presence of *N. gonorrhoeae*.^(12,13)^

Studies on specific high-risk populations also demonstrate a rising prevalence of STIs in the region. Meta-analysis by Davey et al.^(10)^ indicates the Latin-America region (including Peru, Brazil, Ecuador, Argentina, and Guatemala) had the highest adjusted mean prevalence of *C. trachomatis* cases among pregnant women (11.2%), and one of the highest *N. gonorrhoeae* prevalences (1.2%). Vallejo-Ortega et al.^(14)^ also found that Latin-American adolescents have important prevalences rating from 2.1 to 30.1% for *C. trachomatis* and 0 to 2.9% for *N. gonorrhoeae*.

Ecuador, a South American Andean country, implemented the "Plan Estratégico Nacional de VIH," initially known as "Estrategia Nacional de Prevención y Control del VIH/SIDA-ITS." This government strategy focused on controlling human immunodeficiency virus (HIV) and hepatitis A, B, and C infections.^(15-23)^ Unfortunately, other STIs don't have a systematic approach in this setting. Reports on chlamydial and gonococcal diseases are scarce in Ecuador, but the *C. trachomatis* incidence varies from 0% to 41%, while *N. gonorrhoeae* ranges from 0% to 20% (Chart 1). Increasing prevalences are observed among pregnant teenagers and sexual workers (Chart 1).

**Chart 1:** Published studies that report the presence of *Chlamydia trachomatis* and *Neisseria gonorrhoeae* in women populations

First author, year of collection, reference	Women characteristics (number of participants, ages)	*Chlamydia trachomatis*	*Neisseria gonorrhoeae*
Narváez (1989)^(24)^	Sexual workers (116, 15-45)	29%*	20.6%**
Narváez (1989)^(24)^	Promiscuous (136, 15-45)	34.5%*	13.2%**
Narváez (1989)^(24)^	Pregnant (61, 15-45)	0%*	1.6%**
González-Andrade and Aguinaga-Romero (2015)^(25)^	Hospital Admissions/Discharges (all nationally registered)	0.66%***	0.8%***
Medina et al. (2009)^(26)^	Pregnant with threatened preterm labor and preterm premature rupture of membranes (158)	8.2%****	-
Ortiz Segarra et al. (2023)^(27)^	Indigenous women (396, underage and adults)	6.06%****	0.51%****
Vasco et al. (2016)^(28)^	Pregnant teenagers (86, 12-19)	41.8%****	-
Llangarí-Arizo et al. (2021)^(29)^	Sexual workers (249, 18-61)	4.9%****	1.2%****
Rayo O et al. (2017)^(30)^	Adults (200, 18-45)	1.5%****	-
Abad et al. (2022)^(31)^	Asymptomatic (102, 18-45)	2.94%*****	0%*****

* direct immunofluorescence;

** Thayer-Martin culture;

*** Government health care database;

**** Real-time Polymerase Chain Reaction (PCR);

***** PCR and flow-through hybridization

Our study aimed to evaluate the reliability of self-collection and first-catch urine samples compared to cervical brushing samples for *C. trachomatis* and *N. gonorrhoeae* detection in pregnant and non-pregnant adolescents and adult women who sought gynecological services in Quito, Ecuador, by employing automated nucleic acid amplification test (NAAT) cobas^®^ 4800 system for detection.^(16)^

## Methods

We recruited women (assigned female at birth) attending gynecological services in Quito City, Ecuador, from October 2017 to April 2018 who were sexually active. The recruitment occurred at three healthcare facilities: *Hospital Gineco-Obstétrico Isidro Ayora*, a public reference gynecological hospital; *Hospital de Especialidades Carlos Andrade Marín*, a medical specialties hospital under the administration of the *Instituto Ecuatoriano de Seguridad Social*; and *Centro de Salud Cipriana Dueñas*, a public ambulatory clinic from the Obstetricians Carrer at the Universidad Central del Ecuador. The cervical samples were collected by professional gynecologists (at the hospitals) or professional obstetricians (at the ambulatory clinic) who volunteered to participate in the sample collection. The sample size was calculated using a 10% known population incidence, a 5% study incidence,^(6)^ an alfa of 0.05 and a power of 80% gave a minimum sample of 238 participants. We categorized as pregnant and not pregnant and then three age brackets: less than 19 years old, between 19 and 24 years old, and over 24 years old. All pregnant participants were included only if they were less than 20 weeks of gestational age and no contraindications for the cervical speculum examination. Participants were approached at the nursing rooms in the ambulatory services from the hospitals and the clinic before the gynecological consultation. Patients who agreed to participate in the study signed an Informed Consent approved by the Universidad San Francisco de Quito USFQ ethics committee CEISH-USFQ (reference 2016-140M). The investigator conducted a survey that included information regarding sexual behaviors and symptoms of past and present vaginal infections.

Practitioners were provided with detailed instructions for cervical sample collection and patients were provided instructions on how to obtain the first-catch urine and perform the self-collection. We utilized cobas^®^ sample collection kits, which included a tube with a nucleic acid preservative solution for embedding the samples right after the sampling. All samples were collected on the same day. To collect the first-catch urine, the participant was instructed to wash her hands, use a sterile urine collection container, and avoid cleaning the vulva beforehand. The participant had to collect a maximum of 20 ml of first discharged urine to prevent dilution. The urine sample was transferred to the cobas^®^ collection tube (cobas^®^ PCR Urine Sample Kit, P/N: 05170486190). To prepare for the self-collection, the patient was similarly instructed to wash her hands, then open the labia with one hand, and use the other hand to gently introduce the swab into the vaginal canal by approximately 5 cm. The swab had to be softly rubbed against the vaginal walls for about 30 seconds before being removed carefully. The patient was then instructed to open the cap of the cobas^®^ collection tube (cobas^®^ PCR Media Dual Swab Sample Kit, P/N: 07958021190), insert the swab, break it by the swab shaft, and ensure that the cotton bud was embedded in the nucleic acid preservative. Finally, during the gynecological consultation, the practitioner established there were no contraindications for the cervical sampling and proceeded with the following protocol. Firstly, after carefully hand washing, the practitioner used gloves and a sterile speculum to observe the cervix. The practitioner cleaned the cervical area from discharge using a sterile swab. The practitioner used a sterile Rovers^®^ Cervex-Brush^®^ and gave five clockwise rotations into the cervix. The practitioner removed the brush tip and introduced it in a cobas^®^ collection tube (cobas^®^ PCR Cell Collection Media, P/N: 05619637190). All samples were stored at 2-8°C until further analysis was performed. We used the cobas^®^ 4800 System Sample Preparation Kit (P/N: 05235782190) and the nucleic acid amplification test cobas^®^ 4800 CT/NG Amplification/Detection Kit (P/N: 05235952190) for the qualitative detection of *C. trachomatis* and *N. gonorrhoeae*, for all three sample types.

Statistical analyses were performed in JASP software version 0.16.4. We calculated the logistic regression (*X*^2^, *z* value) of the dependent variable positive samples to *C. trachomatis*. We calculated the odds ratio using 95% confidence intervals for any significant finding. The cervical samples taken by a practitioner were set as the gold standard sample type. Sensitivity was defined as the ability of a sample type to correctly identify patients with a disease, and specificity as the ability of a sample type to identify patients without the disease.^(17)^ The sensitivity was calculated as the number of true positives (positives for both the alternative and the gold standard tests) divided by the sum of the true positives and false negatives (positive for the gold standard test but not for the alternative test). The specificity was calculated between the number of true negatives (negative for both the alternative and the gold standard tests) over the sum of the true negatives and false positives (negative for the gold standard test but positive for the alternative test).

## Results

**Figure 1:**
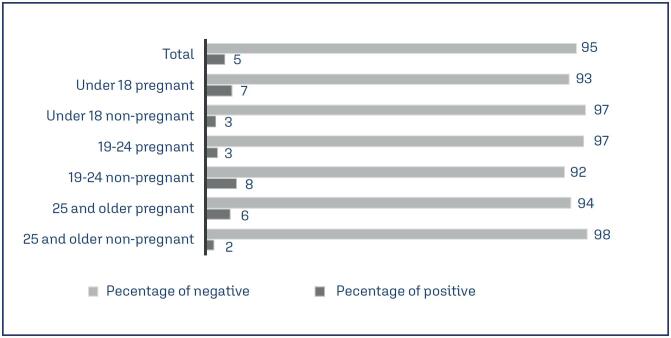
Percentage of patients tested positive or negative for *Chlamydia trachomatis* distributed by age group and pregnancy status

We enrolled 249 participants, of which 124 were pregnant. We obtained at least one sample from 244 participants, including 236 cervical samples, 224 self-collected samples, and 221 urine samples. For the following analysis, we excluded participants without any sample data. The average age of the participants was 22.9 years (±6.15 standard deviation (SD), range 14 to 37 years). Only 12 out of the 244 women tested positive for *C. trachomatis* for at least one sample type with a valid result, representing a 4.92% prevalence (95% CI 2.8 to 8.4%) (Figure 1). Furthermore, all the samples were negative for *N. gonorrhoeae*.

We gathered 209 participants who provided both cervical and self-collected samples with valid results. And 203 participants had one first-catch urine and one cervical sample with a valid result. We observed that for the self-collection versus cervical sample, there were nine true positives and zero false negatives for a performance of 100% sensitivity (95% CI 66.4% to 100.0%) and zero false positives and 209 true negatives for a 100% specificity (95% CI 98.3% to 100.0%). In the first-catch urine versus cervical sample, there were nine true positives and one false negative for a 90% sensitivity (95% CI 55.5% to 99.8%), and two false positives and 201 true negatives for a 99% specificity (95% CI 96.5% to 99.9%). The only variable associated with a negative result for *C. trachomatis* was if patients had been in a stable intimate relationship in the past year (logistic regression χ^2^ = 14.01, *p* < 0.001; OR = 0.092, 95% CI 0.027 to 0.312; z = −3.827 *p* < 0.001, 95% CI −3.613 to −1.166). None of the other clinical variables, such as pregnancy status, the number of sexual partners, the presence of vaginal discharge, symptoms of a vaginal disease, or the use of a contraceptive method were associated with a positive or negative *C. trachomatis* result (Table 1).

**Table 1:** Related sexual behaviors and factors with a positive or negative outcome of *Chlamydia trachomatis*

Variables		*Chlamydia trachomatis* positive n(%) (n=12)	*Chlamydia trachomatis* negative n(%) (n=232)	Logistic regression Value X^2^ (p)
Age				
	Mean (SD)	21.6(4.43)	23(6.2)	19.762(0.656)
	Median (Range)	21(16-28)	22(14-37)	
Age of first sexual experience				
	Mean (SD)	16.88(1.64)	17.06(2.96)	
	Median (Range)	16.5(15-21)	16(11-28)	
Age group				
	25 and older	4(33.33)	89(38.36)	0.169(0.919)
	19-24	4(33.33)	66(28.45)	
	Under 18	4(33.33)	77(33.19)	
Age group and pregnancy status				
	25 and older non- pregnant	1(8.33)	44(18.97)	2.67(0.751)
	25 and older pregnant	3(25.0)	45(19.4)	
	19-24 non-pregnant	3(25.0)	35(15.09)	
	19-24 pregnant	1(8.33)	31(13.36)	
	Under 18 non-pregnant	1(8.33)	36(15.52)	
	Under 18 pregnant	3(25.0)	41(17.67)	
Pregnancy status				
	Non-pregnant	5(41.70)	115(49.57)	0.287(0.592)
	Pregnant	7(58.33)	117(50.43)	
Marital status				
	Free union	3(25)	61(26.29)	6.192(0.103)
	Single	9(75)	120(51.72)	
	Divorce	0(0)	3(1.290)	
	Married	0(0)	48(20.69)	
Race				
	Mestiza	12(100)	219(94.4)	1.247(0.742)
	Indigenous	0(0)	2(0.86)	
	White	0(0)	6(2.59)	
	Afro Ecuadorian	0(0)	4(1.72)	
At a relationship				
	Yes	8(66.67)	194(83.62)	2.145(0.143)
	No	4(33.33)	36(15.52)	
Where have you been in a relationship in the last three months?				
	Yes	4(33.33)	116(50.00)	2.55(0.110)
	No	6(50.00)	61(26.29)	
Have you had a sexual partner recently?				
	Yes	4(33.33)	108(46.55)	0.038(0.846)
	No	2(16.67)	64(27.59)	
Do you have an intimate and stable sexual partner?				
	Yes	5(41.67)	187(80.6)	14.01(< 0.001) *
	No	7(58.33)	24(10.34)	
Have you had different sexual partners in the last year?				
	Yes	4(33.33)	43(18.53)	2.322(0.128)
	No	2(16.67)	79(34.05)	
How many sexual partners have you had in the last year?				
	5	0(0)	1(0.43)	4(0.262)
	3	0(0)	5(2.16)	
	2	3(25.00)	13(5.6)	
	1	8(66.67)	148(63.8)	
Smoke				
	Yes	2(16.67)	12(5.170)	1.906(0.167)
	No	10(83.33)	217(93.53)	
Number of cigarettes by day				
	3	0(0)	2(0.86)	NaN
	2	0(0)	1(0.43)	
	1	0(0)	3(1.29)	
Vaginal shower				
	Yes	8(66.67)	168(72.41)	0.302(0.583)
	No	4(33.33)	59(25.43)	
Number of times daily intimate hygiene				
	3	0(0)	3(1.29)	4.423(0.11)
	2	6(50.00)	52(22.41)	
	1	4(33.33)	132(56.9)	
Vaginal discharge presence				
	Yes	9(75)	162(69.83)	0.103(0.748)
	No	3(25)	67(28.88)	
Vaginal discharge color				
	Green	0(0)	3(1.29)	2.318(0.888)
	Transparent	0(0)	3(1.29)	
	Red	0(0)	2(0.86)	
	Grey	0(0)	5(2.16)	
	Brown	0(0)	3(1.29)	
	White	6(50.00)	101(43.53)	
	Yellow	4(33.33)	46(19.83)	
Pruritus				
	Yes	4(33.33)	65(28.02)	0.422(0.516)
	No	6(50.00)	151(65.09)	
Vaginal discharge has a foul odor				
	Yes	2(16.67)	44(18.97)	0.023(0.880)
	No	8(66.67)	156(67.24)	
Previous vaginal discharges				
	Yes	4(33.33)	129(55.6)	2.553(0.11)
	No	6(50.00)	68(29.3)	
How many times have you received treatment for previous vaginal discharges?				
	4	0(0)	25(10.78)	6.313(0.097)
	3	0(0)	22(9.480)	
	2	3(25)	26(11.21)	
	1	3(25)	45(19.40)	
Discomfort due to vaginal symptoms				
	Yes	2(16.67)	60(25.86)	0.385(0.535)
	No	7(58.33)	129(55.60)	
Period you have had vaginal symptoms				
	Always	1(8.33)	9(3.88)	2.708(0.4390)
	Most of the time	0(0)	20(8.62)	
	Usually	1(8.33)	11(4.74)	
	Occasionally	1(8.33)	23(9.91)	
Have children				
	Yes	5(41.67)	112(48.28)	0.318(0.573)
	No	7(58.33)	112(48.28)	
Number of children				
	3	0(0)	1(0.430)	0.281(0.869)
	2	2(16.67)	35(15.09)	
	1	3(25.00)	80(34.48)	
Contraceptive methods use				
	Yes	4(33.33)	125(53.88)	2.182(0.140)
	No	8(66.67)	102(43.97)	
Hormonal contraceptive use				
	Yes	3(25)	100(43.1)	1.625(0.202)
	No	9(75)	132(56.9)	
Barrier contraceptive use				
	Yes	1(8.33)	34(14.66)	0.424(0.515)
	No	11(91.67)	198(85.34)	
Intrauterine device or tubal ligation				
	Yes	0(0)	7(3.020)	0.717(0.397)
	No	12(100)	225(96.98)	
Regularity for the use of Condon				
	Always	0(0)	13(5.60)	2.131(0.7120)
	Most of the time	1(8.33)	20(8.62)	
	Usually	0(0)	4(1.72)	
	Occasionally	0(0)	20(8.62)	

NaN = not a number;

* significant

## Discussion

In our study, we confirmed that self-collection taken by the patient had the same performance as cervical samples taken by a practitioner in detecting positive or negative results for *C. trachomatis* using cobas^®^ technology in a population with a 5% *C. trachomatis* incidence. Indeed, Lunny et al.^(7)^ concluded that self-collected samples for the diagnosis of *C. trachomatis* were an acceptable alternative in cases where women cannot have a vaginal speculum examination, however, first-catch urine was 10% less sensitive than the cervical sampling. Blake et al.^(8)^ reported a 91.7% sensitivity estimate of the urine samples analyzed with Aptima combo 2 (NAAT) versus GenProbe (nucleic acid hybridization) for *C. trachomatis*, and Ferrero et al.^(18)^ reported 91.2% sensitivity in urine samples from men and women for GenProbe versus cell culture. A reduced sensitivity performance may result from nucleic acid inhibition in the urine, sample dilution, inhibition of the PCR reaction due to urine metabolites, or the absence of the *C. trachomatis* infection in the urinary tract.^(19)^

We did not identify any cases of *N. gonorrhoeae* infection. We attribute this finding to the study's sample size, which may not be sufficient to detect the lower prevalence of *N. gonorrhoeae* compared to *C. trachomatis.* Increasing the sample size could provide a more comprehensive understanding of the prevalence of this infection in the Ecuadorian population.

First-catch urine and self-collection methods offer greater convenience for the patient, as they eliminate the need for direct assistance from a healthcare professional. Particularly, self-collection for the detection of *C. trachomatis*, reduces costs associated with special materials such as the vaginal speculum, as well as the price of the medical consultation.^(8)^ However, even when first-catch urine and self-collection require a proper explanation about ensuring sampling and conservation, they may enable the patient to adhere to testing.^(3)^ Patients who deliver self-taken samples indicate being more comfortable with the sampling,^(3)^ and STIs clinics with protocols that include self-collection tests are more prompted to test STIs’ etiological agents than those with other protocols.^(3)^

Of particular concern is the absence of significant related symptoms in those infected patients, as this may discourage seeking a medical diagnosis or even practitioners to encourage STI screening.^(20,21)^

Healthcare providers should consider chlamydial disease testing for all sexually active individuals, particularly in prenatal care, women during pregnancy, and those previously diagnosed with other STIs such as HIV, syphilis, and gonorrhea.^(21,22)^ Prevention measures include counseling on the consistent use of barrier condoms, regular STI testing to detect re-infections, and reducing the number of sexual partners.^(22)^ The Centers for Disease Control and Prevention (CDC) in the United States has recently recommended Doxi PEP usage, which is post-intercourse administration of a single 200 mg dose of doxycycline.^(23)^ Doxi PEP administration reduces the risk of chlamydia and syphilis transmission in men who have sex with men and transgender women. However, its effectiveness in women is still being evaluated.^(23)^

Prompt treatment of chlamydia is necessary to reduce complications such as pelvic inflammatory disease, infertility, or even neonatal complications.^(22)^ Doxycycline is the recommended first-line treatment in non-pregnant women and men, with azithromycin or levofloxacin considered second-line options.^(22)^ For pregnant women, azithromycin is considered safe, and amoxicillin is an alternative treatment.^(22)^ Erythromycin use, although it is used routinely in other settings, is still an alternative treatment with more adverse effects that may hinder patient compliance.^(22)^ Patients should abstain from sexual activity during antibiotic therapy and for at least seven days after completion. Furthermore, it is essential to treat all sexual partners. Treatment compliance can be challenging, and strategies such as single-dose treatment and direct observation of the first dose administration can help improve compliance.^(22)^

## Conclusion

Our study encourages using self-collection for *C. trachomatis* as an alternative sampling to cervical swabs taken by a practitioner in an Ecuadorian population, regardless of pregnancy status or age, even when the patients report no vaginal discharge or abdominal pain.

## References

[1] Rowley J, Vander Hoorn S, Korenromp E, Low N, Unemo M, Abu-Raddad LJ (2019). Chlamydia, gonorrhoea, trichomoniasis and syphilis: global prevalence and incidence estimates, 2016. Bull World Health Organ.

[2] World Health Organization (WHO) (2022). Global health sector strategies on, respectively, HIV, viral hepatitis and sexually transmitted infections for the period 2022-2030.

[3] National Association of County and City Health Officials (NACCHO) (2021). Implementing express STI services: considerations and lessons learned.

[4] Pettifor A, Walsh J, Wilkins V, Raghunathan P (2000). How effective is syndromic management of STDs? A review of current studies. Sex Transm Dis.

[5] Chaponda EB, Bruce J, Michelo C, Chandramohan D, Chico RM (2021). Assessment of syndromic management of curable sexually transmitted and reproductive tract infections among pregnant women: an observational cross-sectional study. BMC Pregnancy Childbirth.

[6] Kreisel KM, Spicknall IH, Gargano JW, Lewis FM, Lewis RM, Markowitz LE (2021). sexually transmitted infections among US women and men: prevalence and incidence estimates, 2018. Sex Transm Dis.

[7] Lunny C, Taylor D, Hoang L, Wong T, Gilbert M, Lester R (2015). Self-collected versus clinician-collected sampling for Chlamydia and gonorrhea screening: a systemic review and meta-analysis. PLoS One.

[8] Blake D, Maldeis N, Barnes M, Hardick A, Quinn T, Gaydos C (2008). Cost-effectiveness of screening strategies for Chlamydia trachomatis using cervical swabs, urine, and self-obtained vaginal swabs in a sexually transmitted disease clinic setting. Sex Transm Dis.

[9] Van Gerwen OT, Muzny CA, Marrazzo JM (2022). Sexually transmitted infections and female reproductive health. Nat Microbiol.

[10] Davey DJ, Shull HI, Billings JD, Wang D, Adachi K, Klausner JD (2016). Prevalence of curable sexually transmitted infections in pregnant women in low- and middle-income countries from 2010 to 2015. Sex Transm Dis.

[11] Du M, Yan W, Jing W, Qin C, Liu Q, Liu M (2022). Increasing incidence rates of sexually transmitted infections from 2010 to 2019: an analysis of temporal trends by geographical regions and age groups from the 2019 Global Burden of Disease Study. BMC Infect Dis.

[12] Huai P, Li F, Chu T, Liu D, Liu J, Zhang F (2020). Prevalence of genital Chlamydia trachomatis infection in the general population: a meta-analysis. BMC Infect Dis.

[13] Bardach A, Alconada T, Palermo C, Rojas-Roque C, Sandoval MM, Gomez J (2023). Burden of disease of gonorrhoea in Latin America: systematic review and meta-analysis. Infect Dis Ther.

[14] Vallejo-Ortega MT, Gaitán Duarte H, Mello MB, Caffe S, Perez F (2022). A systematic review of the prevalence of selected sexually transmitted infections in young people in Latin America. Rev Panam Salud Publica.

[15] Ecuador. Ministerio de Salud Pública (2024). Informe anual de la situación epidemiológica del VIH Ecuador 2022.

[16] Van Der Pol B, Taylor SN, Liesenfeld O, Williams JA, Hook EW (2013). Vaginal swabs are the optimal specimen for detection of genital Chlamydia trachomatis or neisseria gonorrhoeae using the Cobas 4800 CT/NG Test. Sex Transm Dis.

[17] Swift A, Heale R, Twycross A (2020). What are sensitivity and specificity?. Evid Based Nurs.

[18] Ferrero DV, Meyers HN, Schultz DE, Willis SA (1998). Performance of the gen-probe AMPLIFIED Chlamydia trachomatis assay in detecting Chlamydia trachomatis in endocervical and urine specimens from women and urethral and urine specimens from men attending sexually transmitted disease and family planning clinics. J Clin Microbiol.

[19] Johnson R, Newhall W, Papp J, Knapp J, Black C, Gift T (2002). Screening tests to detect Chlamydia trachomatis and neisseria gonorrhoeae infections --- 2002. MMWR Recomm Rep.

[20] Dela H, Attram N, Behene E, Kumordjie S, Addo KK, Nyarko EO (2019). Risk factors associated with gonorrhea and Chlamydia transmission in selected health facilities in Ghana. BMC Infect Dis.

[21] Mylonas I (2012). Female genital Chlamydia trachomatis infection: where are we heading?. Arch Gynecol Obstet.

[22] Workowski KA, Bachmann LH, Chan PA, Johnston CM, Muzny CA, Park I (2021). Sexually transmitted infections treatment guidelines, 2021. MMWR Recomme Rep.

[23] Bachmann LH, Barbee LA, Chan P, Reno H, Workowski KA, Hoover K (2024). CDC clinical guidelines on the use of doxycycline postexposure prophylaxis for bacterial sexually transmitted infection prevention, United States, 2024. MMWR Recomm Rep.

[24] Narváez M, López Jaramillo P, Guevara A, Izurieta A, Guderian R (1989). Prevalencia de Chlamydia trachomatis y neisseria gonorrhoeae en tres grupos de mujeres ecuatorianas de distinta conducta sexual. Bol Oficina Sanit Panam.

[25] González-Andrade F, Aguinaga-Romero G (2015). Sexual transmitted infections leading to hospitalization in Ecuadorian patients. Rev CIEZT Clín Cir.

[26] Medina M, Moya W, Hidalgo L, Calle A, Terán E, Chedraui P (2009). Molecular identification of endocervical Chlamydia trachomatis infection among gestations at risk for preterm birth in Ecuador. Arch Gynecol Obstet.

[27] Ortiz Segarra J, Vega Crespo B, Campoverde Cisneros A, Salazar Torres K, Delgado López D, Ortiz S (2023). Human papillomavirus prevalence and associated factors in indigenous women in ecuador: a cross-sectional analytical study. Infect Dis Rep.

[28] Vasco G, Jácome P, Masache J, Marcillo J, Arroyo M, Vivero S (2016). Alta prevalencia de Chlamydia trachomatis en adolescentes embarazadas de Quito, Ecuador. Rev Fac Cien Med (Quito).

[29] Llangarí-Arizo LM, Sadiq ST, Márquez C, Cooper P, Furegato M, Zhou L (2021). Sexually transmitted infections and factors associated with risky sexual practices among female sex workers: a cross sectional study in a large Andean city. PLoS One.

[30] Rayo O S, Peralta S A, Baroja O I (2017). Frecuencia de Chlamydia trachomatis en mujeres en edad fértil al usar PCR en tiempo real en el Servicio de Laboratorio del Hospital Carlos Andrade Marín. Cambios Rev Méd.

[31] Abad S, Neira E, Viñansaca L, Escandón S, Neira VA (2022). Prevalence of Chlamydia trachomatis, ureaplasma urealyticum, and neisseria gonorrhoeae in asymptomatic women from urban-peripheral and rural populations of Cuenca, Ecuador. Infect Dis Rep.

